# Supplementary material for: Immune-induced TCR-like antibodies regulate specific T cell response in mice
Source: Nat Commun. 2026 Apr 16;17:3227. doi: 10.1038/s41467-026-71384-1 (PMC13087043; doi:10.1038/s41467-026-71384-1)
Supplement: Supplementary file 1 — Supplementary Information [file 41467_2026_71384_MOESM1_ESM.pdf]

Supplementary Information of

Immune-induced TCR-like antibodies regulate specific T cell response in mice

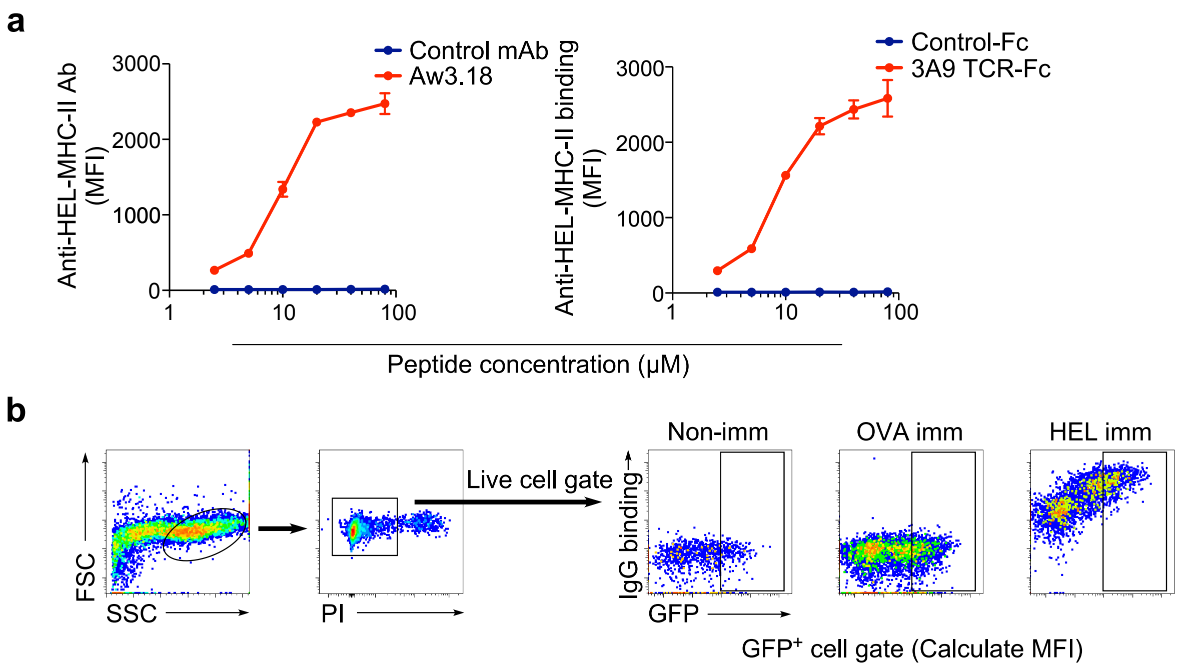

Supplementary Fig. 1: HEL peptide presentation by MHC-II was detected with Aw3.18 and 3A9 TCR-Fc. a MHC-II expressing 293T cells were pulsed with different concentrations of HEL_{48-61} peptide and were stained with Aw3.18 Ab (left) or 3A9 TCR-Fc fusion protein (right). **b** Ab binding to MHC-II expressing 293T cells loaded with HEL_{48-64}. The MFI of GFP^{+} cells was analyzed. Data represent the mean +/- SD (*n* = 3 technical replicates). The experiments were replicated twice.

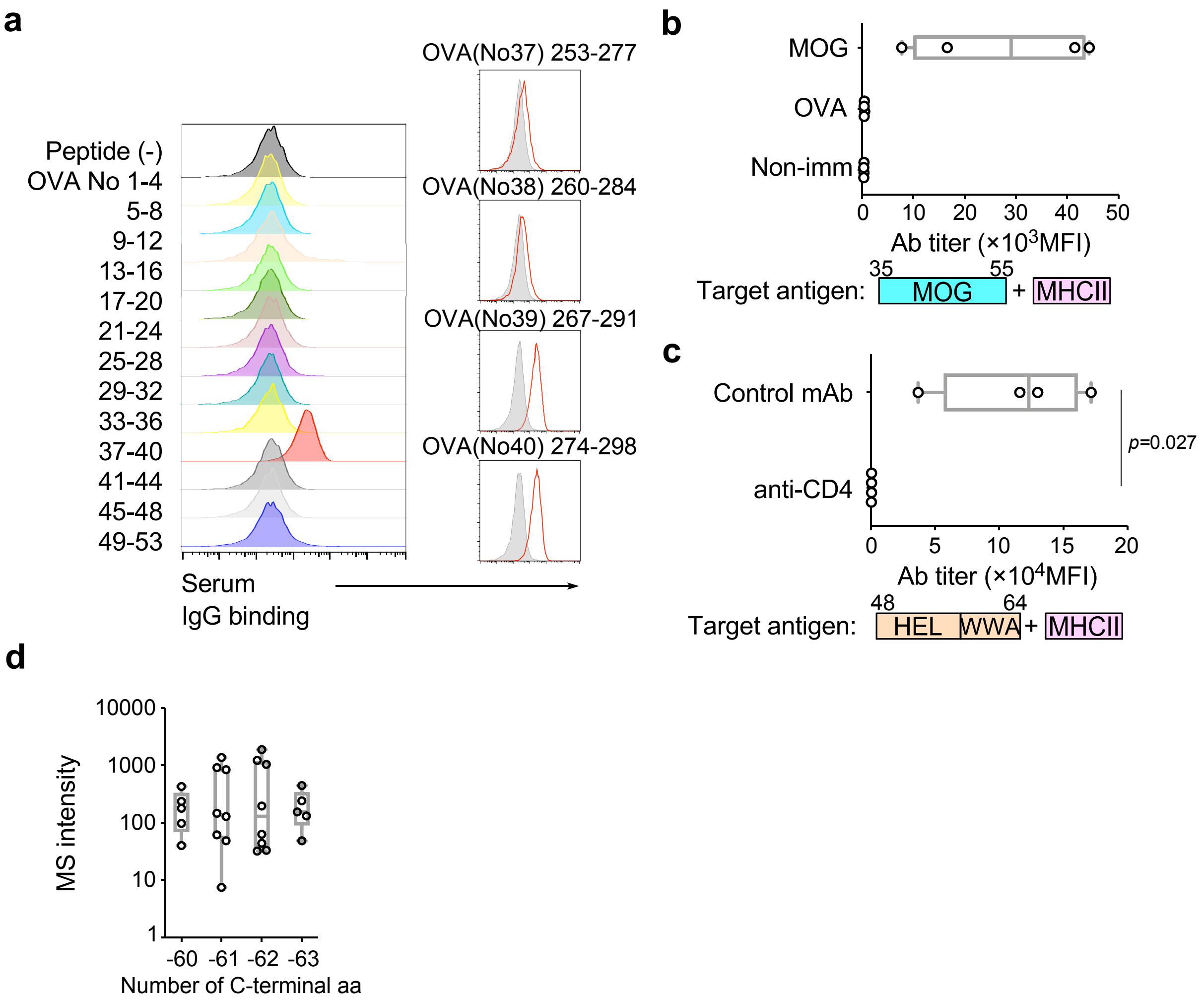

Supplementary Fig. 2: iTab production by antigen immunization and CD4^{+} T cell dependency. a Binding of serum Abs from OVA protein-immunized mice to OVA peptide-pulsed MHC-II expressing 293T cells was analyzed using peptide library. Non-immunized mouse sera were used as controls (shaded histogram). **b** Binding of serum Abs from MOG protein-immunized mice to MOG peptide-pulsed MHC-II expressing 293T cells. **c** CD4^{+} T cell dependent Ab production (b and c, n = 4 mice per group). **d** Detection of FR^{+} HEL peptide from HEL protein pulsed APCs by mass spectrometry. Source data are provided as Supplementary Table 1. p values were determined by a two-sided Student's t-test [c]. The experiments were replicated twice.

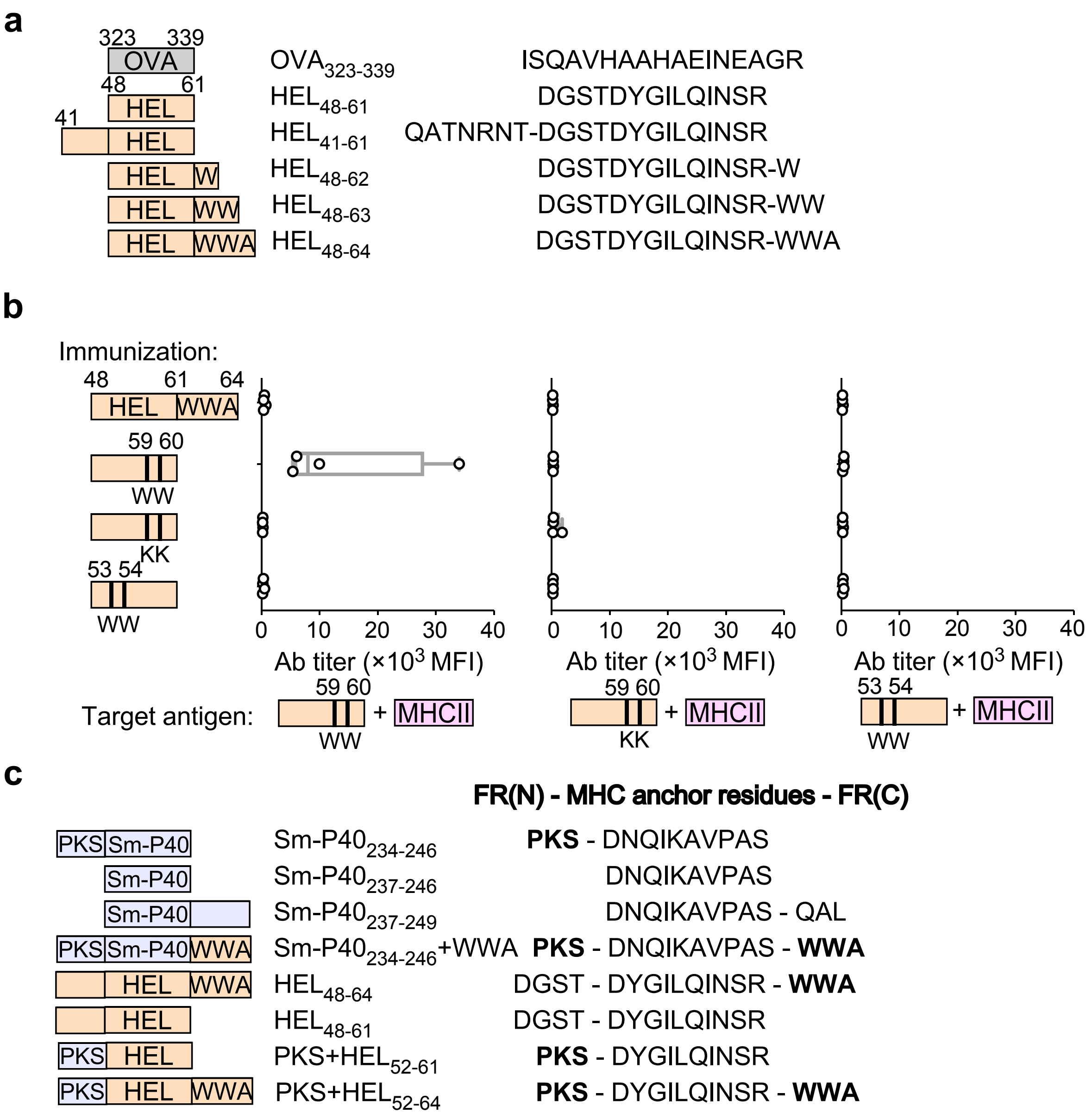

Supplementary Fig. 3: Peptide sequences used in Fig2 and serum iTab titers after mutated FR⁻ peptide immunization.

a Peptide sequence of HEL and control peptide OVA. **b** Serum iTab titers against the mutated FR⁻ HEL peptide/MHCII following immunization with the mutated FR⁻ HEL peptide (n = 4 mice per group). The experiment was replicated twice. **c** Peptide sequence of HEL, Sm-P40 and hybrid peptides. iTab inducible FRs were shown in bold.

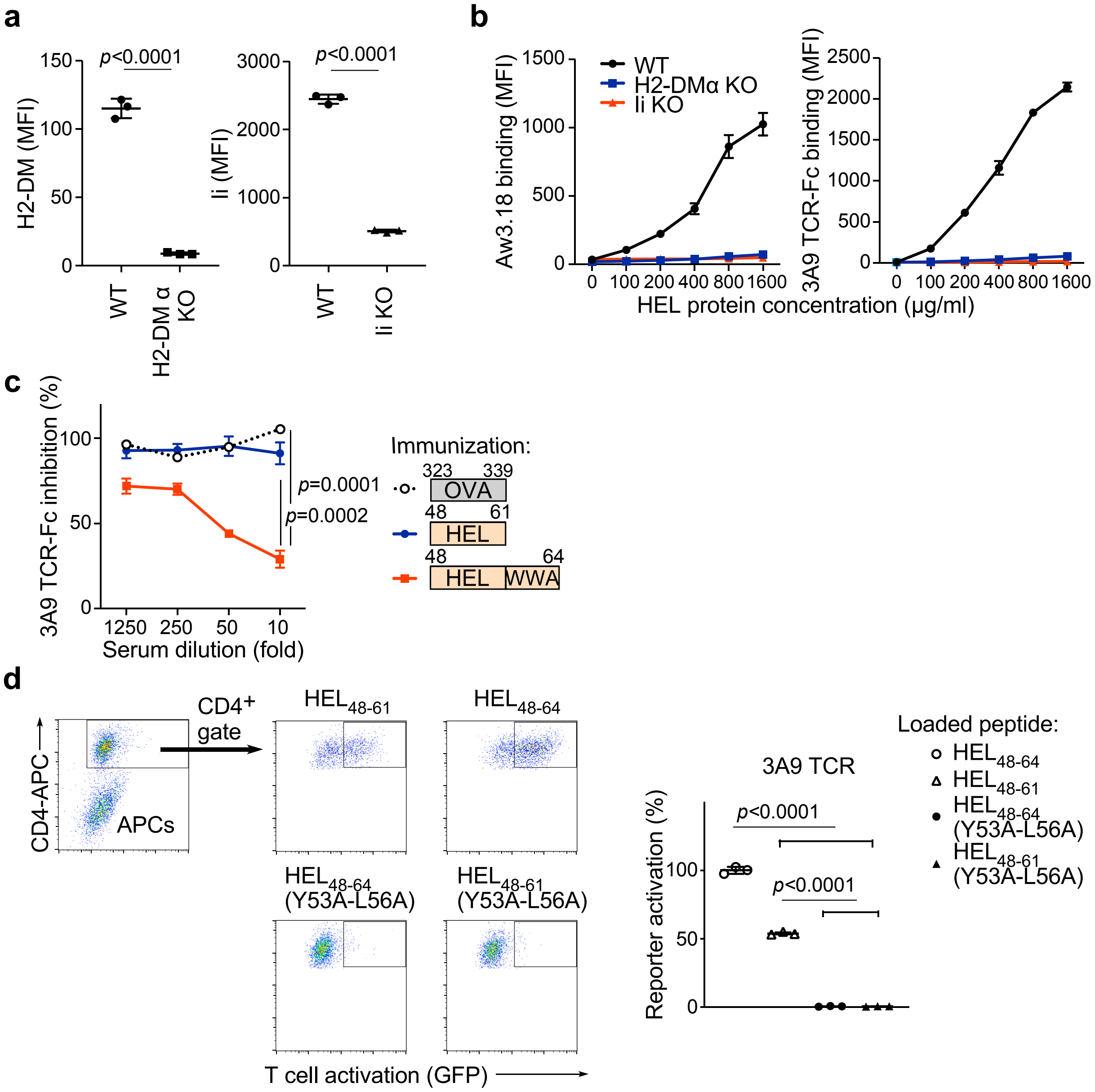

Supplementary Fig. 4: Analysis of Abs in serum immunized with HEL peptides.
a Expression levels of H2-DM or Ii in knockout APCs. **b** Binding of Aw3.18 and 3A9 TCR-Fc against WT LK35.2(black line), H2-DMα (blue line) or invariant chain (red line) knockout cells pulsed with HEL protein. **c** Blocking of 3A9 TCR-Fc binding by Abs induced by immunization with FR^- or FR^+ HEL peptide. **d** Activation of 3A9 TCR-expressing reporter cell with WT and mutated peptide. The percentage of GFP^+ cells when loaded with FR^+ HEL was calculated as 100%. *p* values were determined by one-way ANOVA[d], two-way ANOVA[c] with Tukey's correction, except for [a] where a two-sided Student's *t*-test was used. All data represent the mean +/- SD (*n* = 3 technical replicates). The experiments were performed more than twice.

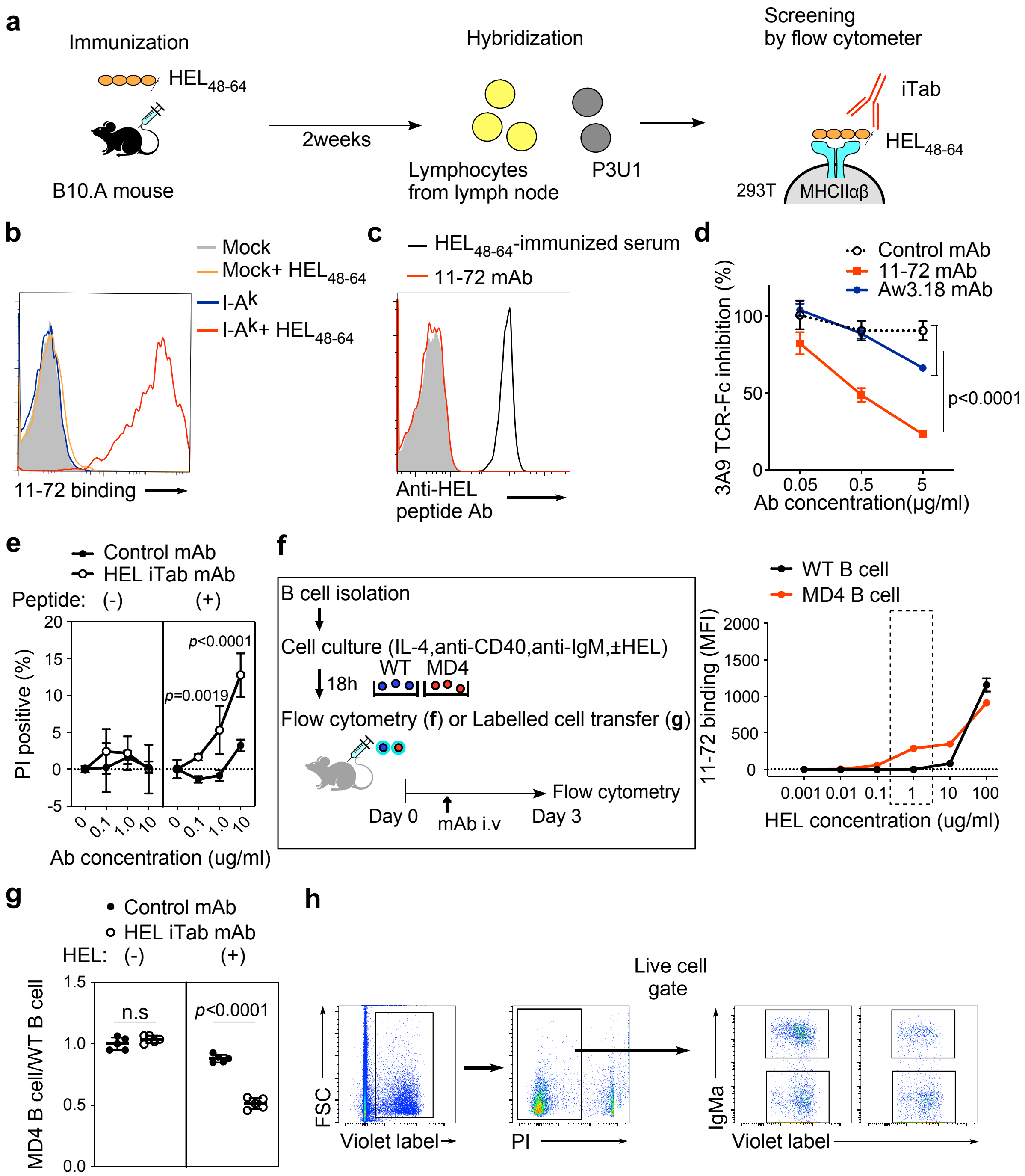

Supplementary Fig. 5: Generation of monoclonal anti-HEL iTab 11-72 and analysis of ADCC mediated by 11-72 mAb.

a Protocol to generate monoclonal anti-HEL iTabs. **b** Binding of anti-HEL iTab 11-72 to HEL_{48-64} peptide-pulsed I-A^k expressing cells. **c** Binding of 11-72 iTab to HEL_{48-64} peptide-coated latex beads. **d** Blocking of 3A9 TCR-Fc binding by 11-72 or Aw3.18 mAb. **e** iTab ADCC activity in vitro. **f** Detection of Antigen presentation in WT and MD4 B cells using 11-72 (d,e and f, $n = 3$ technical replicates). **g** iTab ADCC activity in vivo ($n = 5$ mice per group). **h** The gating strategy of ADCC assay in vivo. p values were determined by two-way ANOVA with Tukey's [d] and Bonferroni's correction [e] except for [g] where a two-sided Student's t-test was used. Data represent the mean +/- SD [d to g]. The experiments were replicated twice.

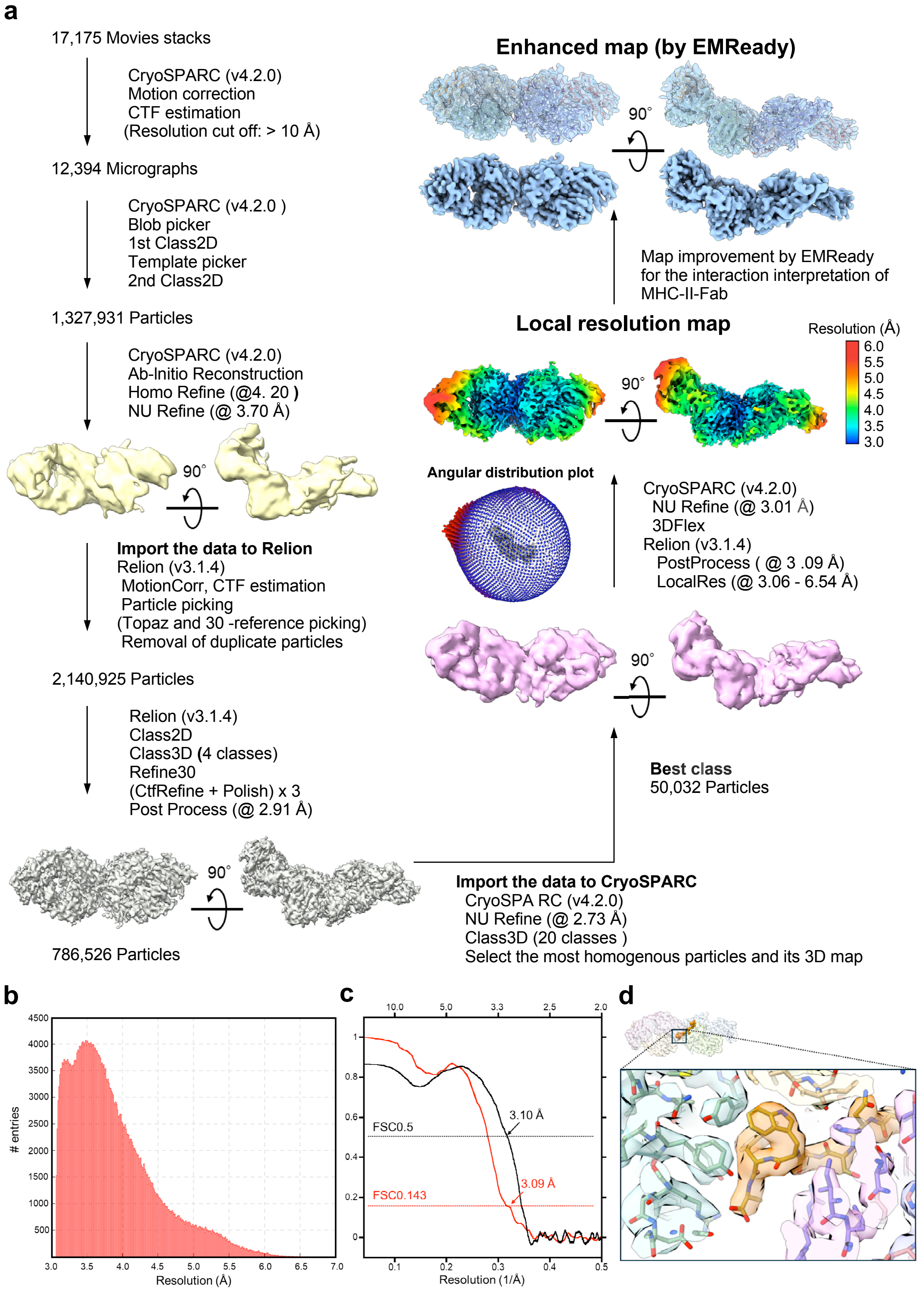

Supplementary Fig. 6: Workflow for data collection and image processing analysis of the anti-HEL iTab and MHC-II complex.

a Overview of the data processing workflow. The resolution was estimated based on the gold standard Fourier shell correlation (FSC) criteria of 0.143. **b** Histogram of local resolution of the 3D map. **c** FSC curves for 3D reconstruction of the 3D map and the refined model versus the overall 3.1 Å map. Red, gold-standard curve with a value of 0.143 at 3.09 Å resolution; black, FSC curve calculated between the 3D map and the refined structure model of the anti-HEL iTab. **d** The enhanced 3D map by EMReady is shown at a contour level of 3.64 σ.

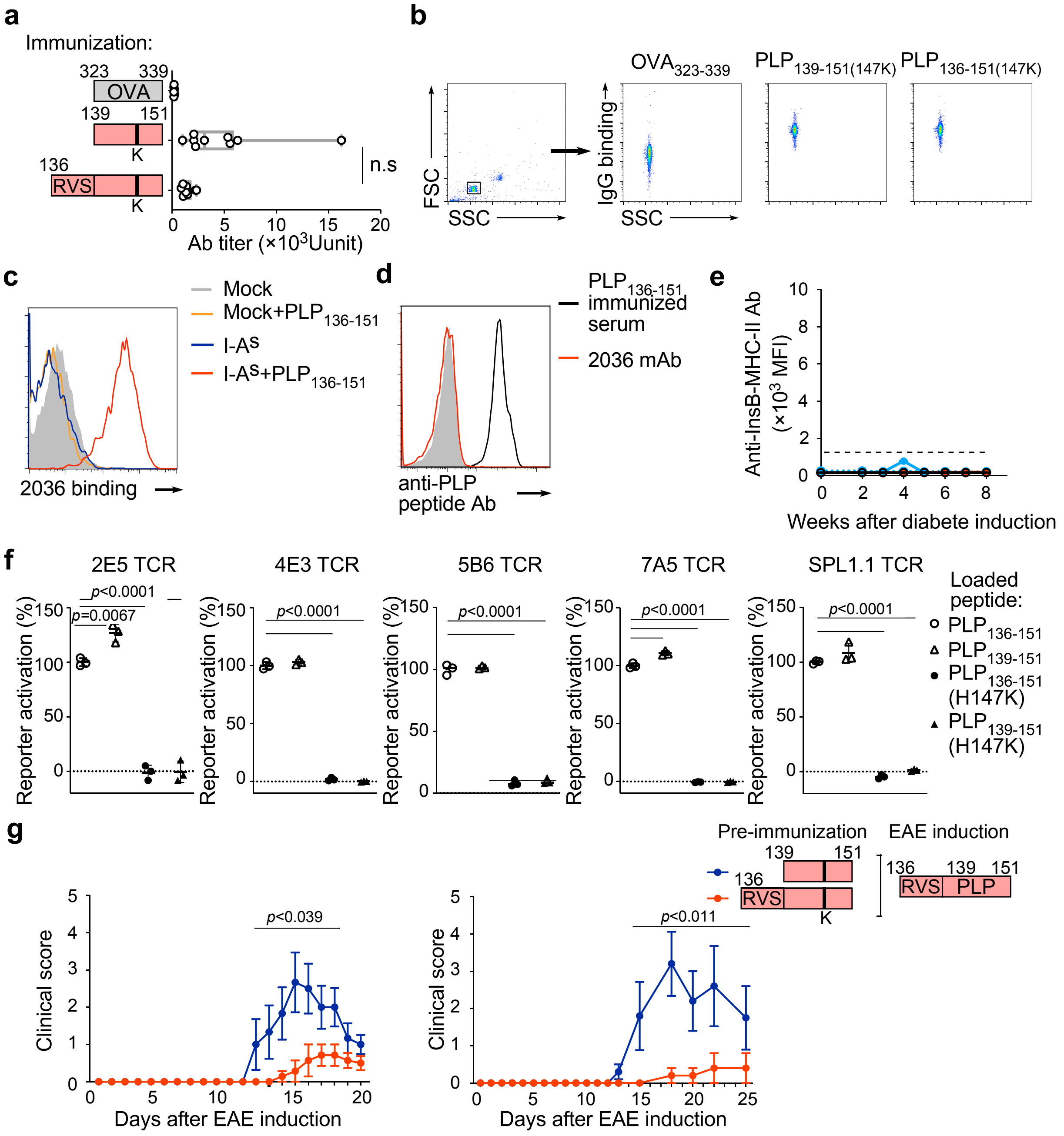

Supplementary Fig. 7: Ab titers of anti-PLP peptide Ab and specificity of monoclonal anti-PLP iTab 2036 and PLP specific-TCR reporter cells.

a Anti-PLP peptide Ab titers in serum immunized with mutated PLP peptide (n = 9 mice per group)**. b** Gating strategy using SA beads fused with biotinylated-PLP$_{136\text{-}151}$ peptide. **c** Binding of anti-PLP iTab 2036 to PLP$_{136\text{-}151}$ peptide pulsed on MHC-II, I-A^s, expressing cells. **d** Binding of 2036 iTab to PLP$_{136\text{-}151}$ peptide-coated beads. **e** iTab titers in diabetes-induced mice (n = 6 mice per group). **f** Activation of PLP-specific TCR reporter cells to WT and mutant PLP peptides (n = 3 technical replicates). **g** Additional experimental data of anti-PLP iTab-inducing peptide immunization ameliorates EAE (n = 5-6 mice per group). p values were determined by one-way ANOVA [a and f] and two-way ANOVA [g] with Bonferroni's correction. Data represent the mean +/- SD [a and f] and +/- SE [g]. The experiments were performed more than twice [a to f].

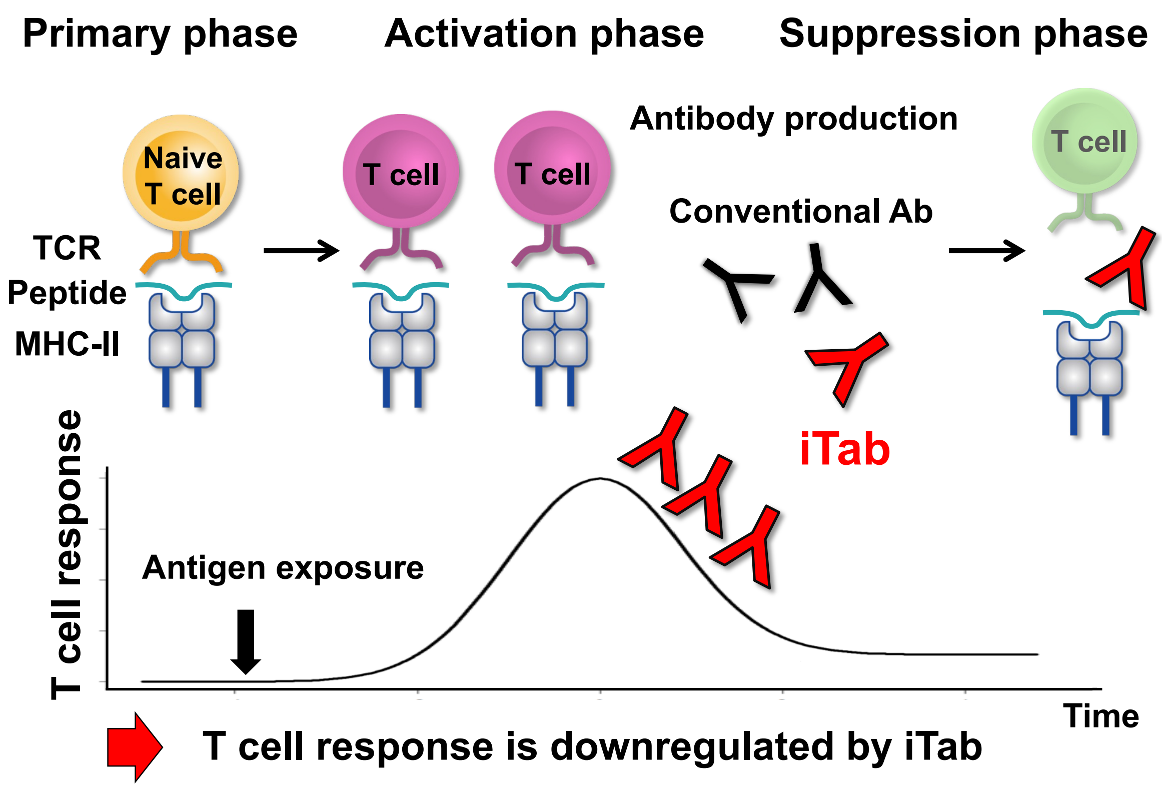

Supplementary Fig. 8: Antigen-specific immune regulation mediated by iTab.

Peptide	Amino acid number		-10lgP	Area
	START	END		
AAKFESNFNTQATN	31	44	55.0	0.00
AAKFESNFNTQATNRN	31	46	28.4	29.14
AKFESNFNTQATNRN	32	46	48.6	0.00
KFESNFNTQATNRN	33	46	38.5	0.00
AAKFESNFNTQATNRNTDG	31	49	57.1	42.15
AAKFESNFNTQATNRNTDGSTD	31	52	59.0	26.43
RNTDGSTDYGILQIN	45	59	61.5	89.14
NTDGSTDYGILQIN	46	59	57.0	19.43
TDGSTDYGILQIN	47	59	52.3	69.49
NRNTDGSTDYGILQINS	44	60	50.9	39.90
NTDGSTDYGILQINS	46	60	45.6	97.05
TDGSTDYGILQINS	47	60	47.9	428.78
DGSTDYGILQINS	48	60	62.2	234.66
GSTDYGILQINS	49	60	63.9	179.48
STDYGILQINS	50	60	34.4	0.00
NRNTDGSTDYGILQINSR	44	61	66.0	7.46
RNTDGSTDYGILQINSR	45	61	62.2	128.02
NTDGSTDYGILQINSR	46	61	71.3	146.32
TDGSTDYGILQINSR	47	61	79.2	918.87
DGSTDYGILQINSR	48	61	74.7	1368.00
GSTDYGILQINSR	49	61	69.1	839.58
STDYGILQINSR	50	61	65.7	61.27
TDYGILQINSR	51	61	61.7	48.64
NRNTDGSTDYGILQINSRW	44	62	67.5	32.05
RNTDGSTDYGILQINSRW	45	62	56.4	32.88
NTDGSTDYGILQINSRW	46	62	68.4	196.02
TDGSTDYGILQINSRW	47	62	82.0	1042.00
DGSTDYGILQINSRW	48	62	77.7	1884.50
GSTDYGILQINSRW	49	62	77.2	1232.30
STDYGILQINSRW	50	62	62.4	62.59
TDYGILQINSRW	51	62	59.4	0.00
YGILQINSRW	53	62	56.4	43.82
NRNTDGSTDYGILQINSRWW	44	63	42.3	0.00
RNTDGSTDYGILQINSRWW	45	63	63.8	154.36
NTDGSTDYGILQINSRWW	46	63	65.8	48.31
TDGSTDYGILQINSRWW	47	63	67.7	131.26
DGSTDYGILQINSRWW	48	63	70.7	443.65
GSTDYGILQINSRWW	49	63	73.4	240.98
YGILQINSRWW	53	63	48.1	0.00

Supplementary Table 1: Peptides presented on MHC-II of APCs co-cultured with HEL protein.

Data collection and processing	anti-HEL iTab (EMB- 62748) (PDB 9L1L)
Microscope	CRYOARM 300
Imaging device	K3 summit
Nominal magnification	x100,000
Voltage (kV)	300
Data collection	SerialEM[34], yoneoLocr[35]
Imaging mode	Counting
Electron exposure (e^{-}/Å^2)	~ 99
Frame per movie	100
Defocus range (μm)	-0.2 – -5.0*
Original pixel size (Å)	0.495
No. of total images sets	17,175
Pixel size or final map	0.99
Symmetry imposed	C1
Initial particle images (no.)	2,659,032
Final particle images (no.)	50,032
Map resolution (Å)	3.09
FSC threshold	0.143
Map sharpening *B* factor (Å^2)	-53.77
**Refinement**	
Initial model used (PDB code)	1IAK, 4HC1
Model resolution (Å)	3.1
FSC threshold	0.5
Model resolution range (Å)	247.5 – 3.09
Model composition	
Non-hydrogen atoms	6,383
Protein residues	800
Ligands	-
B factors (Å^2)	
Protein	175.1
Ligand	-
R.m.s. deviations	
Bond lengths (Å)	0.007
Bond angles (°)	1.241
Validation	
MolProbity score	1.95
Clashscore	8.8
Poor rotamers (%)	0.44
Ramachandran plot	
Favored (%)	92.28
Allowed (%)	7.07
Disallowed (%)	0.65

Supplementary Table 2: Cryo-EM data collection, refinement, and validation statistics.
*Defocus range was estimated by CTFFIND4[38].

	Buried surface area (Å^2)
Antibody : Peptide (All residues)	592.3
Antibody: Peptide (C-terminal WW)	301.4
Antibody: Peptide (excluding the C-terminal WW)	291.0
MHC-II : Antibody	690.3

Supplementary Table 3: The buried surface area values of the antibody interface with the peptide and the MHC-II.

Heavy chain (Chain C)

Antibody sequence number	1	2	3	4	5	6	7	8	9	11	12	13	14	15	16	17	18	19	20	21	22	23	24	25	26	27	28	29	30	31	35
Amino acid residue number	1	2	3	4	5	6	7	8	9	10	11	12	13	14	15	16	17	18	19	20	21	22	23	24	25	26	27	28	29	30	31
Amino acid residue name	D	V	Q	L	Q	E	S	G	P	G	L	V	K	P	S	Q	S	L	S	L	T	C	S	V	T	G	Y	S	I	T	S

Antibody sequence number	36	37	38	39	40	41	42	43	44	45	46	47	48	49	50	51	52	53	54	55	56	57	58	59	63	64	65	66	67	68	69
Amino acid residue number	32	33	34	35	36	37	38	39	40	41	42	43	44	45	46	47	48	49	50	51	52	53	54	55	56	57	58	59	60	61	62
Amino acid residue name	A	Y	Y	W	N	W	I	R	Q	F	P	G	N	K	L	E	W	M	G	Y	I	R	Y	D	G	S	N	N	Y	N	P

Antibody sequence number	70	71	72	74	75	76	77	78	79	80	81	82	83	84	85	86	87	88	89	90	91	92	93	94	95	96	97	98	99	100	101
Amino acid residue number	63	64	65	66	67	68	69	70	71	72	73	74	75	76	77	78	79	80	81	82	83	84	85	86	87	88	89	90	91	92	93
Amino acid residue name	S	L	K	N	R	I	S	I	T	R	D	T	S	K	N	Q	F	F	L	K	L	N	S	V	T	T	E	D	T	A	T

Antibody sequence number	102	103	104	105	106	107	108	109	110	111	112A	112	113	114	115	116	117	118	119	120	121	122	123	124	125	126	127	128
Amino acid residue number	94	95	96	97	98	99	100	101	102	103	104	105	106	107	108	109	110	111	112	113	114	115	116	117	118	119	120	121
Amino acid residue name	Y	Y	C	A	R	A	Y	G	S	S	Y	D	Y	A	M	D	Y	W	G	Q	G	T	S	V	T	V	S	S

Light Chain (Chain D)

Antibody sequence number	1	2	3	4	5	6	7	8	9	10	11	12	13	14	15	16	17	18	19	20	21	22	23	24	25	26	27	28	29	36	37
Amino acid residue number	1	2	3	4	5	6	7	8	9	10	11	12	13	14	15	16	17	18	19	20	21	22	23	24	25	26	27	28	29	30	31
Amino acid residue name	D	I	V	M	T	Q	S	H	K	F	M	S	T	S	V	G	D	R	V	S	I	T	C	K	A	S	Q	D	V	G	T

Antibody sequence number	38	39	40	41	42	43	44	45	46	47	48	49	50	51	52	53	54	55	56	57	65	66	67	68	69	70	71	72	74	75	76
Amino acid residue number	32	33	34	35	36	37	38	39	40	41	42	43	44	45	46	47	48	49	50	51	52	53	54	55	56	57	58	59	60	61	62
Amino acid residue name	T	V	A	W	Y	Q	Q	K	P	G	Q	S	P	K	L	L	I	Y	W	A	S	T	R	H	T	G	V	P	D	R	F

Antibody sequence number	77	78	79	80	83	84	85	86	87	88	89	90	91	92	93	94	95	96	97	98	99	100	101	102	103	104	105	106	107	108	114
Amino acid residue number	63	64	65	66	67	68	69	70	71	72	73	74	75	76	77	78	79	80	81	82	83	84	85	86	87	88	89	90	91	92	93
Amino acid residue name	T	G	S	G	S	G	T	D	F	T	L	T	I	S	N	V	Q	S	E	D	L	A	D	Y	F	C	Q	Q	Y	S	R

Antibody sequence number	115	116	117	118	119	120	121	122	123	124	125	126	127
Amino acid residue number	94	95	96	97	98	99	100	101	102	103	104	105	106
Amino acid residue name	Y	P	T	F	G	G	G	T	K	L	E	I	K

Supplementary Table4: The IMGT numbering scheme of 11-72 monoclonal antibody.

Antigen	Catalogue number	Manufacturer	Final dilution
mouse IgG	715-136-151	Jackson ImmunoResearch Laboratories	1:200
mouse IgG1	A21240	Thermo (Invitrogen)	1:200
human IgG Fc	109-136-098	Jackson ImmunoResearch Laboratories	1:200
mouse CD4	17-0041-83	Thermo (eBioscience)	1:200
H2-DM	552405	BD Biosciences	1:200
Ii	151002	Biolegend	1:200
rat IgG	712-136-153	Jackson ImmunoResearch Laboratories	1:400
mouse IgMa	408614	BioLegend	1:200

Supplementary Table5: List of the antibodies used in this paper.
