# Supplementary material for: Immune-induced TCR-like antibodies regulate specific T cell response in mice
Source: Nat Commun. 2026 Apr 16;17:3227. doi: 10.1038/s41467-026-71384-1 (PMC13087043; doi:10.1038/s41467-026-71384-1)
Supplement: Supplementary file 3 — Reporting Summary [file 41467_2026_71384_MOESM3_ESM.pdf]

nature portfolio

Corresponding author(s): Hisashi Aease

Last updated by author(s): Mar 10, 2026

Reporting Summary

Nature Portfolio wishes to improve the reproducibility of the work that we publish. This form provides structure for consistency and transparency in reporting. For further information on Nature Portfolio policies, see our Editorial Policies and the Editorial Policy Checklist.

Statistics

For all statistical analyses, confirm that the following items are present in the figure legend, table legend, main text, or Methods section.

n/a	Confirmed	
☐	☒	The exact sample size (*n*) for each experimental group/condition, given as a discrete number and unit of measurement
☐	☒	A statement on whether measurements were taken from distinct samples or whether the same sample was measured repeatedly
☐	☒	The statistical test(s) used AND whether they are one- or two-sided<br>*Only common tests should be described solely by name; describe more complex techniques in the Methods section.*
☒	☐	A description of all covariates tested
☐	☒	A description of any assumptions or corrections, such as tests of normality and adjustment for multiple comparisons
☐	☒	A full description of the statistical parameters including central tendency (e.g. means) or other basic estimates (e.g. regression coefficient) AND variation (e.g. standard deviation) or associated estimates of uncertainty (e.g. confidence intervals)
☐	☒	For null hypothesis testing, the test statistic (e.g. *F*, *t*, *r*) with confidence intervals, effect sizes, degrees of freedom and *P* value noted<br>*Give P values as exact values whenever suitable.*
☒	☐	For Bayesian analysis, information on the choice of priors and Markov chain Monte Carlo settings
☒	☐	For hierarchical and complex designs, identification of the appropriate level for tests and full reporting of outcomes
☒	☐	Estimates of effect sizes (e.g. Cohen's *d*, Pearson's *r*), indicating how they were calculated

Our web collection on statistics for biologists contains articles on many of the points above.

Software and code

Policy information about availability of computer code

Data collection	Flow cytometry data was collected using CellQuest or FACSuite software(BD BioScience).<br>Cryo-EM data was collected using SerialEM.<br>Mass spectrometric analysis was performed using timsTOF.
Data analysis	FloJo10 was used for analysis of flow cytometry data.<br>Cryo-EM data were analyzed and processed using cryoSPARC4.2.0 and Relion3.1.4.<br>Graph Pad Prism (Version 10) was used for graphical and statistical analysis.<br>PEAKS Xpro was used for mass spectrometry analysis .

For manuscripts utilizing custom algorithms or software that are central to the research but not yet described in published literature, software must be made available to editors and reviewers. We strongly encourage code deposition in a community repository (e.g. GitHub). See the Nature Portfolio guidelines for submitting code & software for further information.

Data

Policy information about availability of data

All manuscripts must include a data availability statement. This statement should provide the following information, where applicable:

- Accession codes, unique identifiers, or web links for publicly available datasets
- A description of any restrictions on data availability
- For clinical datasets or third party data, please ensure that the statement adheres to our policy

The authors declare that the data supporting the findings of this study are available within the paper, its supplementary files and source data. The structural data generated in this study have been deposited in the Protein Data Bank(PDB) under accession code 9L1L, and cryo-EM maps have been deposited in the Electron Microscopy Data Band(EMDB) under accession codes EMD-62748. The mass spectrometry data have been deposited in the Japan ProteOme Standard Repository (jPOSTrepo) under the accession code JPST004463.

Research involving human participants, their data, or biological material

Policy information about studies with human participants or human data. See also policy information about sex, gender (identity/presentation), and sexual orientation and race, ethnicity and racism.

Reporting on sex and gender	n/a
Reporting on race, ethnicity, or other socially relevant groupings	n/a
Population characteristics	n/a
Recruitment	n/a
Ethics oversight	n/a

Note that full information on the approval of the study protocol must also be provided in the manuscript.

Field-specific reporting

Please select the one below that is the best fit for your research. If you are not sure, read the appropriate sections before making your selection.

[x] Life sciences [ ] Behavioural & social sciences [ ] Ecological, evolutionary & environmental sciences

For a reference copy of the document with all sections, see nature.com/documents/nr-reporting-summary-flat.pdf

Life sciences study design

All studies must disclose on these points even when the disclosure is negative.

Sample size	No statistical methods were used to pre-determine the sample size. Most experiments were repeated at least 2 times to ensure an overall sample size of at least 3 per experimental group. The exact n values used to calculate the statistics are provided per experiment presented in the main and supplementary figure legends of the manuscript.
Data exclusions	No data were excluded from the analysis.
Replication	Number of replicates is indicated in each Figure legend. In general, data presented are the result of at least 2 independent experiments with multiple biological replicates.
Randomization	Mice were housed in the same room and/or isolators in the respective animal facility. Sex- and age-matched animals were used for experiments.
Blinding	The investigators were not blinded during collection of animal serum due to requirements for cage identification and labeling for treatment purpose. Other experiments administering antibodies or peptides were performed blind.

Reporting for specific materials, systems and methods

We require information from authors about some types of materials, experimental systems and methods used in many studies. Here, indicate whether each material, system or method listed is relevant to your study. If you are not sure if a list item applies to your research, read the appropriate section before selecting a response.

Materials & experimental systems

n/a	Involved in the study	
☐	☒	Antibodies
☐	☒	Eukaryotic cell lines
☒	☐	Palaeontology and archaeology
☐	☒	Animals and other organisms
☒	☐	Clinical data
☒	☐	Dual use research of concern
☒	☐	Plants

Methods

n/a	Involved in the study	
☒	☐	ChIP-seq
☐	☒	Flow cytometry
☒	☐	MRI-based neuroimaging

Antibodies

Antibodies used

For flow cytometry antibodies:
Antigen, manufacturer, fluorophore, cat number
- mouse IgG, Jackson ImmunoResearch Laboratories, APC, 715-136-151.
- human IgG, Jackson ImmunoResearch Laboratories, APC, 109-136-098.
- rat IgG, Jackson ImmunoResearch Laboratories, APC, 712-136-153.
- mouse IgG1, Invitrogen, Alexa Fluor 647, A21240.
- mouse CD4, eBioscience, APC, 17-0041-83.
- mouse CD4, BioXcell, BE0003-1,
- mouse MHCII, Biolegend, 205303
- mouse H2-DM, BD Biosciences, 715-136-151.
- mouse Invariant chain, Biolegend, 151002.
- mouse CD40, Biolegend, 102812.
- mouse IgM, Jackson ImmunoResearch Laboratories, 115-006-075.
- mouse IgMa, Biolegend, 408614.

Validation

Commercially available antibodies have been validated by their respective vendors for species reactivity and application in flow cytometry.

Eukaryotic cell lines

Policy information about cell lines and Sex and Gender in Research

Cell line source(s)

293T and LK35.2 cells were obtained from RIKEN cell Bank. Expi293F cells were purchased from Thermo Fisher Scientific. Aw3.18 hybridoma cells and NK-92 cells were purchased from ATCC.

Authentication

The cell lines were obtained commercially and were not authenticated in-house.

Mycoplasma contamination

Each cell line was routinely tested by PCR.

Commonly misidentified lines
(See ICLAC register)

n/a

Animals and other research organisms

Policy information about studies involving animals; ARRIVE guidelines recommended for reporting animal research, and Sex and Gender in Research

Laboratory animals

B10.A, B10.D2, B10.S and C57BL/10 congenic mice, C57BL/6J and Balb/c were purchased from Japan SLC. NOD/SHiJcl mice were purchased from CLEA JAPAN.B10.A(4R) purchased from the Jackson laboratory. SJL/J mice were purchased from Charles RIver Laboratories Japan and maintained under specific pathogen-free (SPF) conditions. MD4 BCR transgenic mice were provided by Prof. Tomonori Kurosaki.

Wild animals

n/a

Reporting on sex

Mice were age- and sex-matched. Female mice were used for all experiments.

Field-collected samples

n/a

Ethics oversight

All animal procedures were performed in accordance with guidelines of the Animal Research Committee of the Research Institute for Microbial Diseases, Osaka University.

Note that full information on the approval of the study protocol must also be provided in the manuscript.

Plants

Seed stocks	n/a
Novel plant genotypes	n/a
Authentication	n/a

Flow Cytometry

Plots

Confirm that:

- [x] The axis labels state the marker and fluorochrome used (e.g. CD4-FITC).
- [x] The axis scales are clearly visible. Include numbers along axes only for bottom left plot of group (a 'group' is an analysis of identical markers).
- [x] All plots are contour plots with outliers or pseudocolor plots.
- [x] A numerical value for number of cells or percentage (with statistics) is provided.

Methodology

Sample preparation	MHC-II transiently transfected 293T cells were co-incubated with peptides and evaluated antibody binding. LK35.2 cells were co-cultured with HEL protein and analyzed antibody binding.<br>Antigen conjugated beads were prepared by mixture of streptavidin coated aldehyde/sulfate latex beads with biotinylated antigen.<br>Intracellular antibody staining of H2-DM and Ii KO cells were fixed and permeabilized using the BD Cytofix/Cytoperm Fixation/ Permeabilization solution kit.<br>The Wild type or MD4 B cells were cultured ex vivo, fluorescently labeled, transplanted into mice, and analyzed three days later.
Instrument	FACSCaliber, FACSVerse (BD Bio Sciences)
Software	Data collection: CellQuest, FACSuite (BD BioSciences)<br>Data Analysis: FlowJo software (Tree Star, Inc.)
Cell population abundance	n/a
Gating strategy	mouse CD4-APC and GFP positive cells were evaluated in reporter assay(supplementary Fig.4d)

- [x] Tick this box to confirm that a figure exemplifying the gating strategy is provided in the Supplementary Information.
